# Supplementary material for: Enhanced subjective performance achievement in wind instrument playing through positive memory recall: effects of sympathetic activation and emotional valence
Source: Front Psychol. 2025 Sep 3;16:1544069. doi: 10.3389/fpsyg.2025.1544069 (PMC12441066; doi:10.3389/fpsyg.2025.1544069)
Supplement: Supplementary file 1 [file Data_Sheet_1.pdf]

Supplementary Material

1 Supplementary Tables

Supplementary Table 1 Estimated Effect Sizes (β) for SD2/SD1 Differences Between Conditions

Condition Comparison	β	*SE*	*t-value*
**Negative − Positive**	−0.31	0.12	−2.57
**No-memory − Positive**	−0.27	0.12	−2.29
**No-memory − Negative**	0.03	0.12	0.29

Note. β coefficients represent semi-standardized effect sizes, where the outcome variable was standardized. *SE* = standard error.
The intraclass correlation coefficient (ICC) was 0.748 for SD2/SD1, indicating the proportion of variance attributable to between-subject differences.

Supplementary Table 2 Estimated Effect Sizes (β) for SD1 Differences Between Conditions

Condition Comparison	β	*SE*	*t-value*
**Negative − Positive**	0.03	0.1	0.28
**No-memory − Positive**	0.36	0.1	3.59
**No-memory − Negative**	0.33	0.1	3.31

Note. β coefficients represent semi-standardized effect sizes, where the outcome variable was standardized. *SE* = standard error.
The intraclass correlation coefficient (ICC) was 0.821 for SD1, indicating the proportion of variance attributable to between-subject differences.

Supplementary Table 3 Fixed Effects Estimates from Linear Mixed-Effects Model Predicting Performance Achievement

Predictor	*Estimate*	*SE*	*df*	*t-value*	*p-value*	*VIF*
**Intercept**	5.47	0.76	37.70	7.20	<0.001*	−
**Condition: No-memory**	0.78	0.34	70.00	2.31	0.024*	1.00
**Condition: Positive**	1.03	0.34	70.00	3.06	0.003*	1.00
**Years of Professional Experience**	0.00	0.02	33.00	0.01	0.989	1.03
**Trait-level Performance Anxiety**	−0.07	0.10	33.00	−0.74	0.463	1.03

Note. This table presents the fixed effect estimates from a linear mixed-effects model examining the influence of condition (reference: Negative), years of professional experience, and trait-level performance anxiety on performance achievement.
Model formula: *Achievement ~ Condition + Experience + Anxiety + (1 | Participant ID)*
Trait performance anxiety was assessed using a 1–9 scale reflecting the level of anxiety participants typically experience before performances. *p*-values are unadjusted. * indicates $p < .05$.
VIF = Variance Inflation Factor; all values < 2 indicate low multicollinearity.

Supplementary Table 4 Pairwise Comparisons of Condition Effects from Linear Mixed-Effects Model Predicting Performance Achievement (Bonferroni-Adjusted)

Contrast	*Estimate*	*SE*	*df*	*t-value*	p_b
**Negative − Positive**	−1.03	0.34	70.00	−3.06	0.010*
**No-memory − Positive**	−0.25	0.34	70.00	−0.74	1.000
**No-memory − Negative**	0.78	0.34	70.00	2.31	0.071

Note.
Linear mixed-effects model: *Achievement ~ Condition + Experience + Anxiety + (1 | Participant ID).*
pb = Bonferroni-adjusted *p*-value. * indicates $p < .05$.

Supplementary Table 5 Fixed Effects Estimates from Linear Mixed-Effects Model Predicting SD2/SD1

Predictor	*Estimate*	*SE*	*df*	*t-value*	*p-value*	*VIF*
**Intercept**	0.96	0.52	33.69	1.87	0.070	–
**Condition: No-memory**	0.03	0.09	70.00	0.29	0.777	1.00
**Condition: Positive**	0.23	0.09	70.00	2.57	0.012*	1.00
**Years of Professional Experience**	0.01	0.01	33.00	0.83	0.411	1.03
**Trait-level Performance Anxiety**	−0.06	0.07	33.00	−0.90	0.376	1.03

Note. This table presents the fixed effect estimates from a linear mixed-effects model examining the influence of condition (reference: Negative), sex, years of professional experience, and trait-level performance anxiety on SD2/SD1.
Model formula: *SD2/SD1 ~ Condition + Experience + Anxiety + (1 | Participant ID)*
Trait performance anxiety was assessed using a 1–9 scale reflecting the level of anxiety participants typically experience before performances. *p*-values are unadjusted. * indicates $p < .05$.
VIF = Variance Inflation Factor; all values < 2 indicate low multicollinearity.

Supplementary Table 6 Pairwise Comparisons of Condition Effects from Linear Mixed-Effects Model Predicting SD2/SD1 (Bonferroni-Adjusted)

Contrast	*Estimate*	*SE*	*df*	*t-value*	p_b
**Negative − Positive**	−0.23	0.09	70.00	−2.57	0.037*
**No-memory − Positive**	−0.21	0.09	70.00	−2.29	0.075
**No-memory − Negative**	0.03	0.09	70.00	0.29	1.000

Note.
Linear mixed-effects model: *SD2/SD1 ~ Condition + Experience + Anxiety + (1 | Participant ID).*
pb= Bonferroni-adjusted *p*-value. * indicates $p < .05$.

Supplementary Table 7 Fixed Effects Estimates from Linear Mixed-Effects Model Predicting SD1

Predictor	*Estimate*	*SE*	*df*	*t-value*	*p-value*	*VIF*
**Intercept**	−3.46	3.93	32.49	−0.88	0.384	−
**Condition: No-memory**	1.92	0.58	70.00	3.31	0.002*	1.00
**Condition: Positive**	−0.16	0.58	70.00	−0.28	0.782	1.00
**Years of Professional Experience**	0.11	0.11	33.00	1.00	0.323	1.03
**Trait-level Performance Anxiety**	−0.73	0.55	33.00	−1.34	0.188	1.03

Note. This table presents the fixed effect estimates from a linear mixed-effects model examining the influence of condition (reference: Negative), sex, years of professional experience, and trait-level performance anxiety on SD1.
Model formula: *SD1 ~ Condition + Experience + Anxiety + (1 | Participant ID)*
Trait performance anxiety was assessed using a 1–9 scale reflecting the level of anxiety participants typically experience before performances. *p*-values are unadjusted. * indicates $p < .05$.
VIF = Variance Inflation Factor; all values < 2 indicate low multicollinearity.

Supplementary Table 8 Pairwise Comparisons of Condition Effects from Linear Mixed-Effects Model Predicting SD1 (Bonferroni-Adjusted)

Contrast	*Estimate*	*SE*	*df*	*t-value*	p_b
**Negative − Positive**	0.16	0.58	70.00	0.28	1.000
**No-memory − Positive**	2.08	0.58	70.00	3.59	0.002*
**No-memory − Negative**	1.92	0.58	70.00	3.31	0.005*

Note.
Linear mixed-effects model: *SD1 ~ Condition + Experience + Anxiety + (1 | Participant ID)*.
pb= Bonferroni-adjusted *p*-value. * indicates $p < .05$.
